# Compressional-Wave Effects in the Operation of a Quartz Crystal Microbalance in Liquids:Dependence on Overtone Order

**DOI:** 10.3390/s20092535

**Published:** 2020-04-29

**Authors:** Robert Kowarsch, Yuriy Suhak, Lucia Cortina Eduarte, Mohammad Mansour, Frederick Meyer, Astrid Peschel, Holger Fritze, Christian Rembe, Diethelm Johannsmann

**Affiliations:** 1 Institute of Electrical Information Technology, Clausthal University of Technology, Leibnizstr. 28, D-38678 Clausthal-Zellerfeld, Germany; kowarsch@iei.tu-clausthal.de (R.K.); mansour.mohammad.atef@tu-clausthal.de (M.M.); rembe@iei.tu-clausthal.de (C.R.); 2 Institute of Energy Research and Physical Technologies, Clausthal University of Technology, Am Stollen 19B, D-38640 Goslar, Germany; yuriy.suhak@tu-clausthal.de (Y.S.); lucia.cortina.eduarte@tu-clausthal.de (L.C.E.); holger.fritze@tu-clausthal.de (H.F.); 3 Institute of Physical Chemistry, Clausthal University of Technology, Arnold-Sommerfeld-Str. 4, D-38678 Clausthal-Zellerfeld, Germany; frederick.sebastian.meyer@tu-clausthal.de (F.M.); peschel@pc.tu-clausthal.de (A.P.)

**Keywords:** quartz crystal microbalance, QCM, thickness-shear resonator, vibration analysis, laser-Doppler vibrometry, flexural motion, compressional waves

## Abstract

The operation of the quartz crystal microbalance (QCM) in liquids is plagued by small flexural admixtures to the thickness-shear deformation. The resonator surface moves not only in the transverse direction, but also along the surface normal, thereby emitting compressional waves into the liquid. Using a simple analytical model and laser Doppler vibrometry, we show that the flexural admixtures are stronger on the fundamental mode than on the overtones. The normal amplitude of motion amounts to about 1% of the transverse motion on the fundamental mode. This ratio drops by a factor of two on the overtones. A similar dependence on overtone order is observed in experiments, where the resonator is immersed in a liquid and faces an opposite planar wall, the distance of which varies. Standing compressional waves occur at certain distances. The amplitudes of these are smaller on the overtones than on the fundamental mode. The findings can be rationalized with the tensor form of the small-load approximation.

## 1. Introduction

The operation of acoustic resonators (of the quartz crystal microbalance, QCM, in particular) in liquids became commonplace after 1985 when Bruckenstein et al. [1] and Nomura et al. [2,3] combined a QCM with electrochemistry. (There are scattered reports of earlier attempts in that direction [4,5]). Soon later, numerous applications in the life sciences followed [6,7].

In hindsight, these researchers were lucky, because there were excellent reasons to expect that the resonance frequency and the resonance bandwidth of a QCM operated in a liquid would be close to useless with regard to interpretation.

The liquid-phase QCM as such had actually crossed people’s minds as early as 1949, when Ma-son, McSkimin, and others published an article reporting on how they had used AT-cut quartz plates to extend the frequency range of their instrumentation upwards to a few MHz [8]. At the time, there was a community of researchers pushing forward acoustic sensing in liquids, mostly using centimeter-sized resonators operating at kHz frequencies (often cylindrical rods). The knowledge achieved in these days is summarized in Mason’s book from 1948 [9]. When turning to MHz frequencies, Mason and co-workers did *not* directly immerse the thickness-shear resonators into the liquid [8]. Rather, they glued them to the ends of cylindrical rods of fused quartz. The crystals launched transverse acoustic waves into the rod. The waves travelled down the rod, were reflected at the other end (which was immersed in the liquid), and returned to the piezoelectric crystal, which also served as the detector. The viscosity of the liquid was derived from the reflected amplitude. In the introduction, Mason et al. expand on why the cylindrical rod was needed. They write: “*Consideration was given to the use of a thickness vibrating shear crystal of the AT or BT type, but it was found that the shear motion was too closely coupled to other modes of motion, such as flexure modes, to give reliable results. Hence another method had to be used*”.

Today, we know from experience that thickness-shear crystals of the AT-type *can* be used as such in liquids. They are employed routinely and work fine, basically. The worries of Mason et al. were largely unjustified, although their line of reasoning certainly makes sense.

It is worthwhile to briefly remind oneself of the problem. In liquids, shear sound and longitudinal sound are much different matters. Longitudinal sound (compressional waves) propagates, as known from ultrasonic imaging. Shear sound, on the other hand, decays within about one wavelength; the wave is strongly damped. Because the shear wave’s depth of penetration (at MHz frequencies) amounts to about a hundred nanometers, the QCM is *surface-specific*, which is one of its prime advantages as a sensor. Surface specificity is lost, when the resonator emits compressional waves in addition to shear waves. Compressional waves propagate, are reflected somewhere, and return to the crystal [10,11,12]. Because the entire liquid cell takes part in this process, compressional waves are not easily controlled.

Unrelated to the decay depth is the wave impedance, *Z*, which is the ratio of stress to particle velocity. As long as the small-load approximation applies [13], the stress-velocity ratio at the resonator surface governs the shifts of frequency and bandwidth. For plane waves propagating inside a homogeneous medium, *Z* is a materials parameter given as

(1) $$Z=\sqrt{K\rho }.$$

ρ is the density and *K* is a modulus. The wave impedance is a key variable in the interpretation of QCM measurements. When immersed into a semi-infinite homogeneous medium, the complex frequency shift, Δ*f* + i ΔΓ (with *f* the resonance frequency and Γ the half-bandwidth), is given as

(2) $$\frac{\Delta f+i\Delta \Gamma }{f_{0}}=\frac{i}{\pi \;Z_{q,eff}}Z.$$

*f*_0_ is the frequency of the fundamental mode and *Z_q,eff_* is the shear-wave impedance of the resonator (or some effective parameter close to that, in case flexural motion is taken into account). Again, Equation (2) only holds for semi-infinite media (no reflections, no waves returning to the crystal). Furthermore, Equation (2) builds on the small-load approximation. As discussed in Section 6.1.3 in Reference [13], the small-load approximation does not necessarily apply to flexural motion.

Compressional waves and shear waves are governed by different moduli, which are the “P-wave modulus” and the “shear modulus”, respectively. The P-wave modulus, *M* (also: “longitudinal modulus”), is much larger than the shear modulus, *G*, because liquids are nearly incompressible, while they are sheared easily. The shear modulus is given as *G* = i ω η, with η being the viscosity. Typical values for |*M*| and |*G*| in water at MHz frequencies are 1 GPa and tens of kPa, respectively. Since the compressional-wave impedance is much larger than the shear-wave impedance, even small amounts of flexural admixtures to a thickness-shear vibration should, in principle, cause large normal oscillatory stresses and large values of Δ*f* and ΔΓ, in consequence. Following this argument, Mason and coworkers discarded AT-cut resonators as probes for liquid viscosity.

Mason and McSkimin knew about the flexural admixtures to the vibration pattern of a QCM. These originate from energy trapping [14,15]. In order to let the amplitude of the shear vibration be zero at the rim of the plate (a condition needed for mounting the crystal without overdamping it), the resonator is made to be thicker in the center than at the edge. One may picture the resonator as an acoustic lens, where the reflections at the concave surfaces focus the acoustic wave to the center. The details are more complicated, but the lens-picture captures the essence of energy trapping. Importantly, the crystal *bends* in response to the gradient in shear amplitude between the center and the edge. Bending implies normal displacements of the resonator surface.

Today, there is ample experimental evidence of compressional-wave effects [10,11,12,16,17,18]. Many studies rely on coupled resonances, which are caused by planar walls located opposite to the resonator surface, giving rise to standing compressional waves. We are not aware of any such study, where these effects would have been compared between different overtones. We report on such a comparison in Section 5.

For a number of reasons, compressional waves are less detrimental to QCM measurements than Mason and McSkimin believed:

- The amplitudes of the compressional waves are weaker than one might think. Even at the fundamental mode (where they are strongest), they amount to less than 5% of the transverse amplitude in air.
- On the overtones (meaning on the higher eigenmodes, which contain more than one nodal plane) the flexural contributions are weaker than on the fundamental mode.
- The presence of a liquid may decrease the flexural contributions.

Quantitative studies of flexural admixtures to the modes of vibration are somewhat demanding. The earliest investigations go back to the 1960’s. Sauerbrey himself (who discovered the Sauerbrey equation) recognized the problem and reported on an extended study in 1967 [19]. A summary of early attempts can be found in a book chapter by Spencer [20]. Williamson collects the state of the art as of 1990 in [21]. At the time, x-ray topographic imaging (“Lang topography” in Williamson’s words) was a prominent technique. There is an array of indirect methods giving access to the mode shapes. Some of them rely on the accumulation of small particles (including vesicles) at the nodes of vibration, where the amplitude of normal motion is smallest [22,23]. The scanning electrochemical microscope can also visualize a QCM’s amplitude distribution [24]. These studies reveal an amazing complexity. In the late 1990s, laser interferometry (also: “laser-Doppler vibrometry”, LDV) became the prime technique to study vibrating resonators of various kinds [25,26]. Pioneering work with regard to the application to the QCM was reported by Watanabe and co-workers [27] exploiting image-processing techniques and, also, by Brown and co-workers [28] using holography.

There is no lack of attempts to predict flexural motion analytically [29,30,31] or numerically [32]. The analytical treatments all make rather severe approximations. Furthermore, the irregular mode shapes (varying from crystal to crystal) suggest that imperfections play some role. When quantitatively modeling the 3D motion of a crystal—ultimately aiming at an improved model for the QCM as a sensor—one will have to base these models on experiment. Presumably, such an analysis will need input from LDV for every single crystal—which can be done, as we show below.

LDV translates a modulation of the optical path length into a phase modulation of the reflected (or scattered) laser light making use of the laser-Doppler effect. Applying a heterodyne detection scheme, the motion of the surface at the laser spot can be reconstructed. Frequencies may range up to a few gigahertz [33]. Most instruments resolve amplitudes down to the femtometer regime (at 1 Hz bandwidth), which is much below the amplitudes under study here. Note, however, that this sensitivity is only achieved for normal motion. Transverse motion is a more demanding target because it requires LDV measurements from oblique angles. These must rely on scattering rather than reflection. LDV under oblique angles was used in this work to assess the ratios of normal motion and transverse motion on the basis of optical measurements, alone.

This work studies the magnitude of flexural motion in air, in liquids, and as a function of overtone order. Because the flexural contributions are strong on the fundamental mode, compressional-wave effects are strong as well. These problems are known to the practitioner. QCM data from the fundamental mode are often discarded from the analysis because these data are poorly reproducible and difficult to understand (without taking compressional waves into account).

In Section 2, we describe a simplified model, which explains why compressional waves are weaker on the overtones than on the fundamental mode. More specifically, the model predicts that the normal displacement (referenced to the transverse displacement) should scale as 1/*n*^2^ with *n* the overtone order. Section 3 describes the LDV instruments and Section 4 reports the results from LDV. Section 5 describes a separate experiment undertaken with a QCM immersed in a liquid, which quantitatively assesses the influence of compressional waves. Section 6 concludes.

## 2. Flexural Contributions are Most Pronounced on the Fundamental Mode

**Figure 1:**
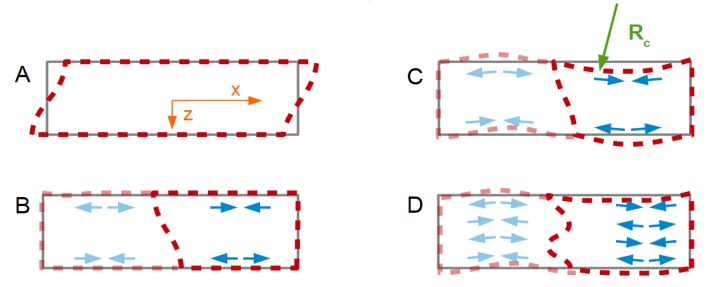
In the idealized parallel plate model, the amplitude of the shear wave is the same, everywhere (**A**). If the amplitude of shear varies across the surface (**B**), a compressive stress and a tensile stress result at the two surfaces of the plate. These will bend the plate (**C**). Bending will also occur on the overtones, but to a lesser extent (**D**).

The argument starts out from the parallel-plate model (shear deformation only, Figure 1A). Furthermore, the argument is based on a quasi-static situation. The transverse displacement, *u_T_*(*z*) is given as

(3) $$u_{T}\left(z\right)={\left(-1\right)}^{(n+1)/2}U_{T}\sin\left(\frac{n\pi z}{2h}\right).$$

*U_T_* is a prefactor to this function. The index *T* stands for transverse motion. The origin of the *z*-scale is in the center of the plate. *h* is half the plate’s thickness. *n* is an odd integer. This form of the equation applies to the odd modes of vibration (*n* = 1,3,5,…). Only the odd modes can be excited piezo-electrically. The sign ensures that the displacement always occurs into the direction of positive *x* at the upper surface. There is no third dimension (the model describes a beam, not a plate.)

Now we switch on energy trapping (Figure 1B) and let the amplitude be a function of *x*. For circular resonator plates, the distribution of the transverse amplitude is often modeled as a Gaussian (large in the center, decaying towards the rim [34]). We assume a linear dependence of *u_T_*(*x*,*z*) on *x* for simplicity (proportional to *U_T_* (1 − *x/L*)). Furthermore, we only consider one half of the bar (to the right in Figure 1B). We write

(4) $$u_{T}\left(x,z\right)={\left(-1\right)}^{(n+1)/2}U_{T}\;\left(1-\frac{x}{L}\right)\;\sin\left(\frac{n\;\pi \;z}{2\;h}\right).$$

The parameter *U_T_* now is to be understood as the maximum transverse displacement amplitude. *L* is half the length of the bar. The factor *U_T_* (1 − *x/L*) produces a lateral gradient in the amplitude of shear and, in consequence, an extensional strain ε*_xx_*, and a corresponding stress σ*_xx_*. Given that the stress has opposite sign at the bottom and the top, there is a *bending* moment (Figure 1C), given as

(5) $$\begin{array}{cc} M_{xy} & =W\int_{-h}^{h}z\;\sigma_{xx}\;dz=W\int_{-h}^{h}z\;\frac{E}{1-\nu }\varepsilon_{xx}\;dz\\ & =W\int_{-h}^{h}z\;\frac{E}{1-\nu }{\left(-1\right)}^{(n+1)/2}\;U_{T}(-\frac{1}{L})\sin\left(\frac{n\;\pi \;z}{2\;h}\right)dz\\ & =W\frac{E}{1-\nu }\frac{8\;h^{2}}{n^{2}\;\pi^{2}}\frac{U_{T}}{L}. \end{array}$$

*W* is the width of the beam. *E*/(1 − ν) is the biaxial modulus, *E* is the Young’s modulus, and ν is Poisson’s number.

If the calculation were to be carried out dynamically, the bending moment would act as a source term in a partial differential equation. For simplicity, we proceed with the quasi-static argument (neglecting inertia).

The beam will bend in response to the gradient in transverse displacement. Let the radius of curvature be *R_c_*. The extensional strain along *x* is

(6) $$\frac{\partial u_{T}}{\partial x}={\left(-1\right)}^{(n+1)/2}\frac{U_{T}}{L}\sin\left(\frac{n\;\pi \;z}{2\;h}\right)-\frac{z}{R_{c}}.$$

The last term (−*z*/*R_c_*) covers curvature. The elastic energy contained in the extensional strain, *W_el_*_,*ext*_, is

(7) $$W_{\mathrm{el},\mathrm{ext}}=\frac{1}{2}\frac{E}{1-\nu }\;L\int_{-h}^{h}{\left[\frac{\partial u_{T}\left(x,z\right)}{\partial x}\right]}^{2}dz=\frac{1}{2}\frac{E}{1-\nu }\;L\int_{-h}^{h}{\left[{\left(-1\right)}^{(n+1)/2}\frac{U_{T}}{L}\sin\left(\frac{n\;\pi \;z}{2\;h}\right)-\frac{z}{R_{c}}\right]}^{2}dz.$$

Using that *n* is an odd integer and carrying out the integration, one finds

(8) $$\frac{1-\nu^{2}}{E}\frac{2\;W_{el,ext}}{L}=\frac{2\;h^{3}}{3}\frac{1}{R_{c}^{2}}+\frac{16\;h^{2}}{\pi^{2}\;n^{2}\;L}\frac{U_{T}}{R_{C}}+\frac{h}{L^{2}}\;U_{T}^{2}.$$

The system will choose the curvature such that the elastic energy is minimized. The derivative of the elastic energy, *W_el_*_,*ext*_, with respect to $R_{c}^{-1}$ must vanish:

(9) $$0=\frac{d}{dR_{c}^{-1}}\left(\frac{2\;W_{el}}{E\;L}\right)=\frac{4\;h^{3}}{3\;R_{c}}+\frac{16\;h^{2}}{\pi^{2}\;n^{2}}\frac{U_{T}}{L}.$$

Solving for the curvature $R_{c}^{-1}$ leads to

(10) $$\left|\frac{1}{R_{c}}\right|=\frac{1}{h}\frac{12}{\pi^{2}\;n^{2}}\frac{U_{T}}{L}.$$

The normal component to the amplitude of motion (which is of the order of *L*^2^/*R_c_*) scales as the curvature and therefore scales as 1/*n*^2^ (Figure 1D). The 1/*n*^2^-scaling is the consequence of the bending moment being proportional to 1/*n*^2^. It would also have been found had the calculation been carried out dynamically (searching the modes of vibration).

The quasi-static model predicts that the transverse motion and normal motion should have similar amplitudes (use *U_N_*~*L*^2^/*R_c_* and insert *R_c_* from Equation (10)). Experiment, on the contrary, shows that the amplitude of normal motion amounts to a few percent of the transverse motion. The shortcomings of this model are the following:

- The quasi-static calculation misses inertial forces, which will reduce the amplitude of normal motion.
- The model treats a bar rather than a cylindrical plate.
- The characteristic lateral scale *L* decreases with overtone order because energy trapping becomes more effective [35].

## 3. Laser-Doppler Vibrometry

Laser-Doppler vibrometry (LDV) exploits the laser-Doppler effect [36]. A laser beam is reflected (or scattered) from a vibrating surface. The displacement phase-modulates the wave in proportion to the change in optical path length. The degree of phase modulation is determined with heterodyne interferometry and is converted to a complex amplitude of displacement *u*(*x*,*y*), where *u* is the projection of the displacement vector onto the optical axis of the beam [37]. For a full characterization of the sample’s motion in 3D, separate LDV experiments from different directions are needed.

In a first set of experiments, LDV was applied to normal motion only Applied to normal motion of optically smooth surfaces, LDV relies on reflection. The normalization to transverse amplitude (needed for assessing compressional-wave effects) occurred on the basis of the electrical current into the electrodes and known relations between current and displacement (Table A1 in Appendix B). In a second set of experiments (using a different LDV instrument), LDV was undertaken from two different angles (Figure A3 in Appendix E). From the projection of the displacement onto these two different axes, one can estimate the ratio of normal to transverse motion from LDV data alone (avoiding the conversion from electric current to transverse amplitude).

This first setup has better accuracy. It was used for comparison between overtones (5 MHz, 15 MHz, and 25 MHz), resonator diameters (14 mm and 25.4 mm), and environments (air and water). Measurements “in liquid” were undertaken with the liquid on the *rear* side. When the cell is mounted this way, the laser beam does not pass through the liquid phase, which makes the optics simpler.

**Figure 2:**
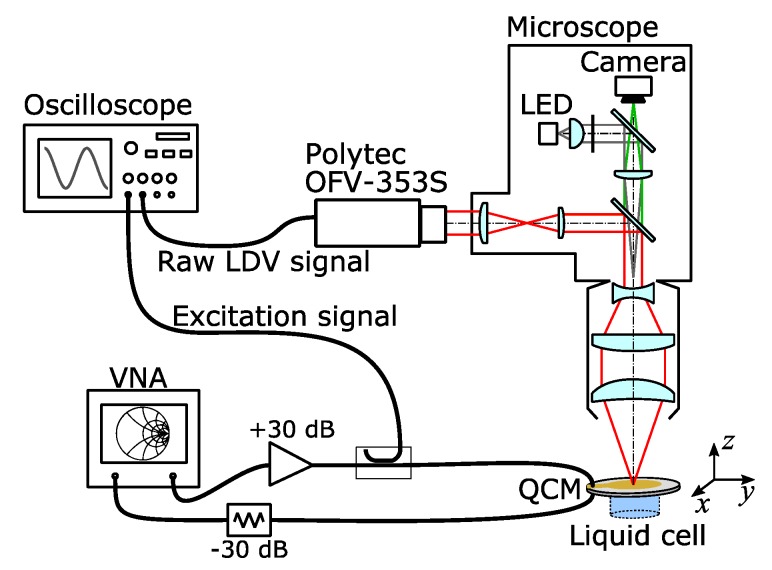
Sketch of the LDV microscope used to study the normal motion of the QCM surface. The unit OFV-353S is a laser-Doppler vibrometer supplied by Polytec (Waldbronn, Germany).

A sketch of the setup is shown in Figure 2. The resonators were driven with a vector network analyzer (S5065, Copper Mountain Technologies, Indianapolis). The network analyzer also determines the electrical current, which allows to estimate the amplitude of transverse displacement. The calculation of transverse displacement requires the motional resistance *R*_1_ (see Appendix B). *R*_1_ was determined with a separate setup. Because the wiring in this other setup was not strictly the same as the wiring in the LDV setup, these amplitudes are reported with single digits in Table 1. One needs to keep in mind that these estimated transverse amplitudes pertain to “equivalent parallel plates” (no energy trapping). Still, the analysis is perfectly valid for comparison between overtones and between air or water.

In preparatory experiments, the excitation frequency was swept across the resonances. The amplitude of normal motion as determined with LDV and the electrical current into the electrodes were both plotted versus frequency. Both plots showed Lorentzians with similar resonance parameters (center frequency and width). We do not further elaborate on these data.

**Figure 3:**
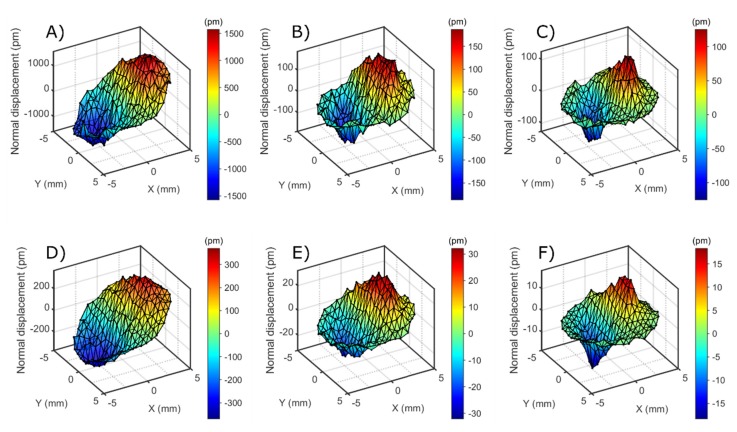
Operating deflection shape in normal direction (diameter of plate: 14 mm). (**A**–**C**): in air. (**D**–**F**): rear side in contact with water; Frequencies: (**A**,**D**): 5 MHz, (**B**,**E**): 15 MHz, (**C**,**F**): 25 MHz.

The maps shown in Figure 3 were acquired by driving the resonator at one single frequency close to the center of the resonance. The drive level was +15 dBm. The laser beam remained fixed in space; the resonator in its holder (home-built) was scanned relative to the beam with a translation stage. Certain measures were taken to extend the frequency range of this setup to 25 MHz (the frequency of the highest overtone studied.) By default, this unit is limited in frequency to 3 MHz by the decoder [38]. In order to circumvent this problem, the raw LDV signal was digitized and demodulated by IQ demodulation (in-phase-and-quadrature demodulation [36]). Spectral filtering at the known excitation frequency suppressed noise and other interferences. The amplitude resolution of normal displacement was 0.5 pm (at 4 Hz bandwidth).

**Figure 4:**
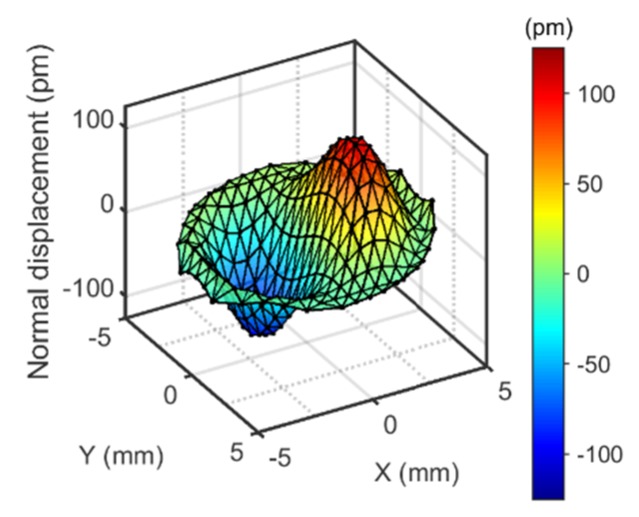
The operating deflection shape obtained after Zernike filtering the raw ODS from Figure 3C. The extracted amplitude is 120 pm (see Table 1).

**Figure A1:**
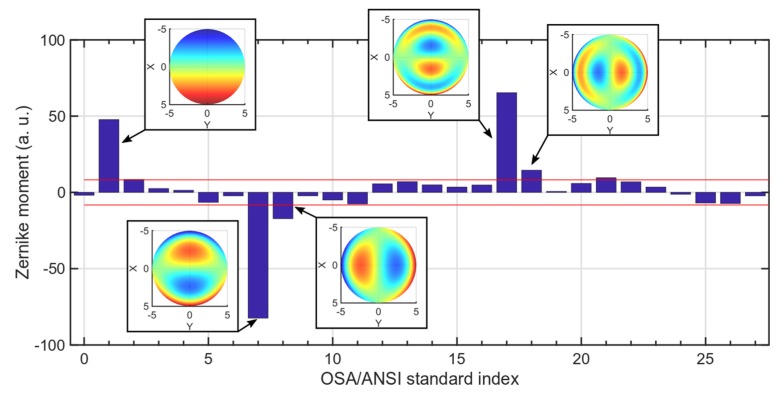
A Zernike decomposition of the operating deflection shape shown in Figure 3C. The dominating mode carries index 7.

The operating deflection shape (ODS) was derived from the amplitudes acquired at about 250 spots separated from each other by 0.6 mm. The raw data occasionally contained spikes, presumably caused by dust. These were removed by threshold-based spatial median filtering. This ODS was decomposed into Zernike modes (Figure A1 in Appendix C). In most cases, the Zernike mode 7 (OSA/ANSI indices) was dominant. “Zernike filtering” entailed two steps. Firstly, all modes with an index larger than 78 were discarded. These are the high-frequency modes, which contain substantial amounts of noise. Furthermore, modes were discarded, which had an amplitude of less than 10% of the maximum amplitude (red lines in Figure A1 in Appendix C). Often, these modes had a symmetry, which did not match the symmetry of the problem. Figure 4 shows an example of a Zernike-filtered ODS. The peak of the Zernike-filtered ODS was named *U_N_*. The parameter *U_N_* served for comparison between overtones and between different experimental configurations.

**Figure A3:**
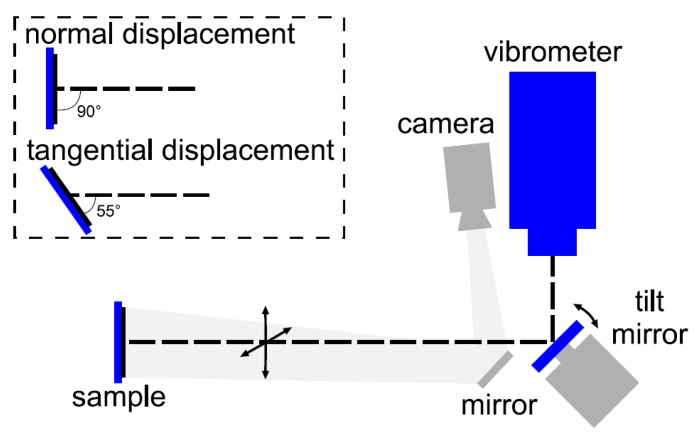
Sketch of the setup used to determine both the normal and the transverse component of the displacement from LDV.

LDV measurements under two different angles were undertaken with the setup described in [39] (different from the instrument sketched in Figure 2). This system is based on the unit OFV-505 from Polytec (Waldbronn, Germany). A sketch is shown in Figure A3 of Appendix E. Spatial maps were acquired using a tip/tilt-mirror, which scanned the beam. A crystal with a diameter of 25.4 mm and a fundamental frequency of 3 MHz was studied. The resonator was coated with a layer of tin (~500 nm) by laser ablation. This layer scatters well enough to allow for LDV measurements based on scattered light. We assume this layer to take little influence on energy trapping. The instrument reports the absolute values of the amplitudes of motion, but it does not report phases. Maps of these absolute values can be produced, but the determination of the full ODS would require the phases. An estimation of the transverse amplitudes from LDV measurements from oblique angles is still feasible if the parasitic normal displacements are much smaller than the transverse displacements.

The transverse motion is expected to occur along the crystallographic *x*-direction. For the examined crystal with a diameter of 25.4 mm and a fundamental frequency of 5 MHz, this direction was independently determined with optical conoscopy as described in Section 13.5 in Reference [13]. Briefly, the resonator is inserted into a polarizing microscope. A further lens (“Bertrand lens”) is introduced between the pupil and the camera, which creates an image of the pupil (rather than an image of the object itself) on the camera. Positions on this image correspond to *angles*, under which the light passes through the crystal (hence the name “conoscopy”). Crossed polarizers produce a ring-shaped pattern, the center of which is the crystallographic *z*-axis (the optical uni-axis). The *x*-axis is in the plane of the crystal (such is the crystal cut) and is tangential to the ring pattern at the center of the field of view.

## 4. Results from Laser-Doppler Vibrometry

### 4.1. LDV Under Normal Incidence

Figure 3 shows the operating deflection shapes (ODS) at the first odd overtones obtained with the setup from Figure 2. In order to extract numbers from these maps (to be compared between the different experimental settings, Table 1), the maps were Zernike-filtered as described in Section 3. The maximum of the filtered ODS was termed *U_N_*.

The deflection shape does not vary much between overtones and between different resonators. Bending is strong where the gradient of the displacement is collinear with the displacement itself. The extrema are displaced from the center along the crystallographic *x*-axis. The gradient is strong at locations about halfway between the center and the rim. The distance of the peak from the center of the plate decreases with the increasing overtone order (Table 2). This is expected, based on the widely accepted understanding of energy trapping. Energy trapping becomes more efficient with increasing overtone order because the key parameter in energy trapping is the ratio of the back-electrode’s thickness to the wavelength of sound. As the latter decreases, the width of the amplitude distribution becomes narrower and the peaks in the gradient of *u_T_*(*x*,*y*) move inwards.

**Figure 5:**
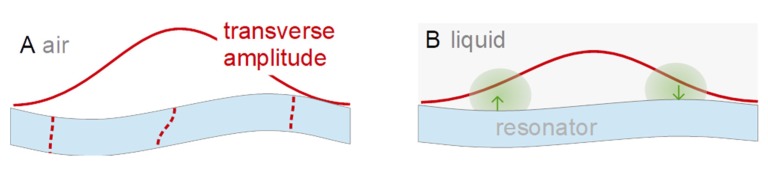
In liquids, the pressure exerted by the fluid reduces bending. There is extensional strain inside the crystal (causing bending) but the same extensional strain is present in the adjacent liquid as well. For volume conservation, extensional strain along *x* inside the liquid causes a corresponding strain along z, which exerts a normal stress onto the plate and thereby *reduces* bending.

Table 1 summarizes the results of this analysis in quantitative form. The following aspects de-serve comments:

- The *U_N_*/*U_T_*-ratio is smaller at 15 MHz (*n* = 3) than at 5 MHz (*n* = 1). However, the 1/*n*^2^-scaling predicted by the model from Section 2 is not quantitatively confirmed. The *U_N_*/*U_T_*-ratio decreases between *n* = 1 and *n* = 3, but it does not decrease by a factor of 9. Comparing *n* = 3 and *n* = 5 (15 MHz and 25 MHz), the *U_N_*/*U_T_*-ratio does not even decrease.
- The *U_N_*/*U_T_*-ratio is larger for the larger crystal (with a diameter of 25.4 mm). While this is not expected, in principle (in-plane gradients decrease when the diameter of the plate increases), one needs to keep in mind that the thickness and the shape of the back electrode also plays a role in energy trapping.
- The *U_N_*/*U_T_*-ratio decreases when the resonator is immersed in water. This is to be expected based on the argument sketched in Figure 5. Bending reduces the extensional strain inside the crystal, but it also causes a pressure in the adjacent liquid. Given that water is nearly incompressible (compared to air), the pressure is substantial and reduces the bending. This argument invalidates the small-load approximation (Section 6.1.3 in Reference [13]). The small-load approximation implicitly claims the modes shape to be unaffected by the load. A side remark: The *U_N_*/*U_T_*-ratio might actually change sign when immersing the resonator into the liquid. The pressure exerted by the liquid might outweigh the consequences of the extensional stress inside the plate. The sign is *not* inverted here. This result contradicts reference [40] (the reasons being unclear).

### 4.2. LDV Under Oblique Incidence

**Figure 6:**
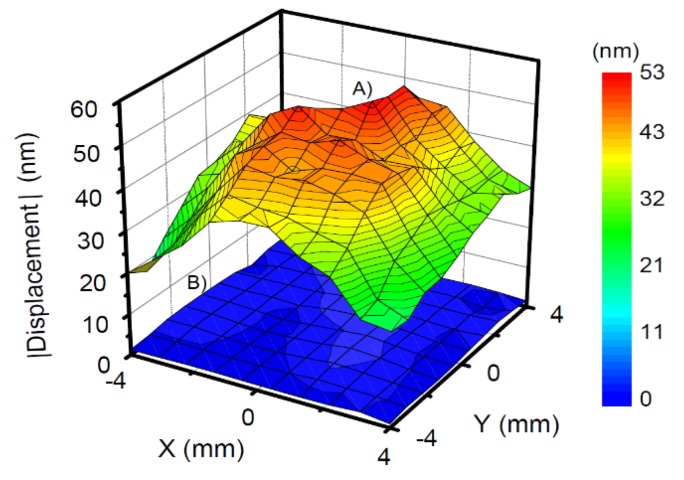
Maps of the absolute values of the amplitudes of motion determined with an LDV setup based on scattering. (**A**) Transverse motion, (**B**) normal motion.

Using the setup allowing for LDV measurements under oblique angles, maps of the absolute values of the ODS were acquired under angles of 55° and 90° (see Figure A3 in Appendix E). (A correction factor of cos (55°) is applied, see Appendix E). These moduli for tangential and normal motion are shown as maps in Figure 6. The maxima in Figure 6A,B are 52 nm and 4 nm, respectively. Even without an explicit reconstruction of the ODS, one can compare magnitudes. The absolute values of the normal motion (Figure 6B) are smaller than the absolute values of the transverse motion (Figure 6A) by at least a factor of 10.

## 5. Cuvette Resonances

The magnitude of compressional-waves effects in a genuine QCM setting was assessed with a well-known and time-honored experimental configuration, namely the resonator plate immersed in a liquid and facing an opposite planar wall, the distance of which varies. To the best of our knowledge, such experiments were first reported by Martin and Hager in 1989 [16]. Whenever the path length of the compressional wave travelling back and forth between the two surfaces equals an integer multiple of the wavelength, constructive interference creates a large amplitude and concomitantly large shifts of frequency and bandwidth. This phenomenon amounts to a coupled resonance, termed “cuvette resonance” here.

**Figure 7:**
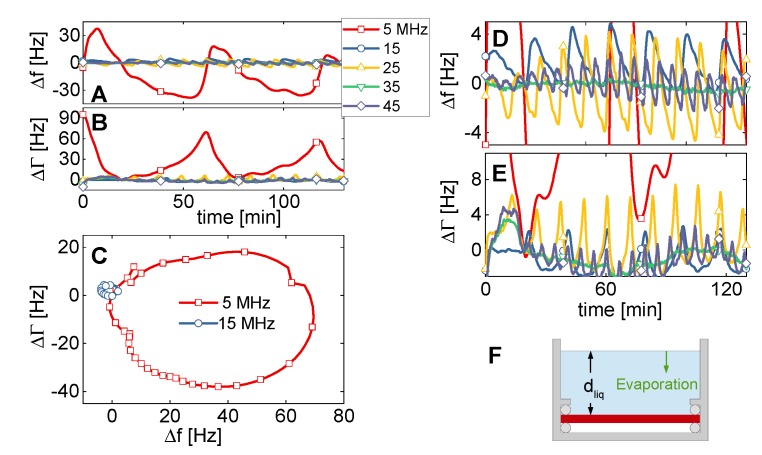
When mounting the resonator horizontally in an open cell (**F**) and letting the liquid evaporate, one observes coupled resonances (**A**–**C**). The vertical enlargement (**D**,**E**) shows the coupled resonances on the overtones. Because of the reduced wavelength, these are more densely spaced than the coupled resonances on the fundamental mode. Furthermore, and more importantly, they occur at a much-reduced amplitude.

A resonator with a diameter of 14 mm was chosen for this experiment (cf. columns 5–7 in Table 1). As the opposing surface, we chose the air-water interface (sketched in Figure 7F). The cell was open, allowing for evaporation. Data were collected overnight. Figure 7A,B) show Δ*f* and ΔΓ versus time for the lower overtones. The key result is evident: Compressional-wave effects are much smaller on the overtones than on the fundamental mode (fundamental: red line, squares). This result alone is reason enough to discard data from the fundamental mode for experiments in liquids (as is common practice). For comparison: The shifts in frequency and bandwidth caused by the liquid’s viscosity amount to about ∓700 Hz. A film with a thickness of 1 nm decreases the resonance frequency by about 6 Hz. A deviation of 30 Hz (as in Figure 7A) is substantial. Figure 7D,E show a vertically expanded plot, in which the data obtained at 5 MHz go off-scale, but data from the higher overtones can be well discerned. The time between successive coupled resonances decreases with overtone order, as can be expected because the wavelength scales as the inverse overtone order.

Figure 7C shows the data from Figure 7A,B as a polar diagram (ΔΓ versus Δ*f*). Circles are found, which is characteristic of coupled resonances [41]. The diameters of the circles in the polar diagram (which count as a measure of the strength of the compressional-wave effect) are provided in Table 3. The circle at 15 MHz is smaller than the circle at 5 MHz by a factor of 12. This comes closer to the 1/*n*^2^-scaling (Section 2) than the *U_N_*/*U_T_*-ratios from Table 1. Similar to Table 1, the radii of the circles in the polar diagram *increase* when going from 15 MHz to 25 MHz. Note that there is no straightforward quantitative relation between the *U_N_*/*U_T_*-ratio and the magnitude of the coupled resonance. They are correlated, but the details involve integrations over the area of the plate. We do not elaborate on these.

**Figure 8:**
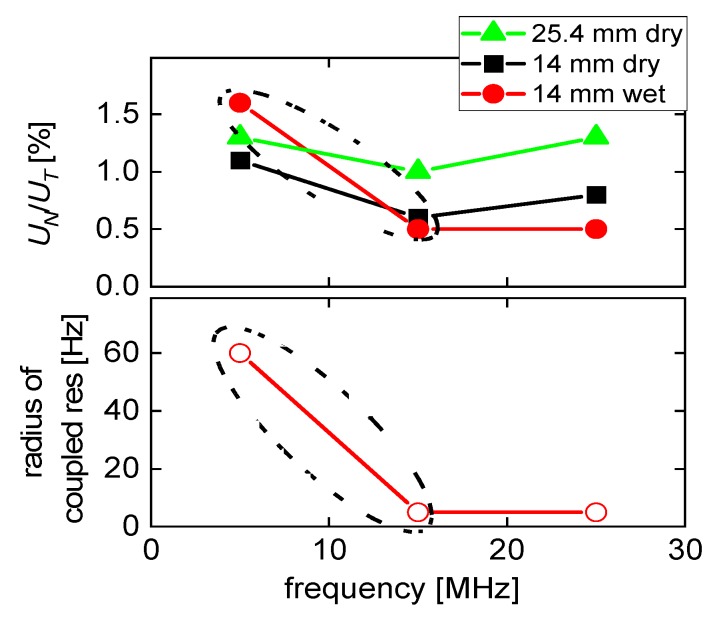
Results from Table 1 and Table 3 displayed in graphical form. *U_N_*/*U_T_*-ratios and the radius of the circle in the polar diagram (Figure 7C) both decrease when going from 5 MHz to 15 MHz.

Figure 8 summarizes the essential results from Table 1 and Table 3 in graphical form. Most importantly, both the *U_N_/U_T_*-ratio and the radii of the circles in Figure 7C decrease when going from the fundamental mode to the third overtone. That decrease does not continue when going to the 5th overtone at 25 MHz, though. This finding is in contrast with results from measurements in the dry, where effects of flexural contributions can also be found. We elaborate on these experiments in Appendix D.

**Figure 9:**
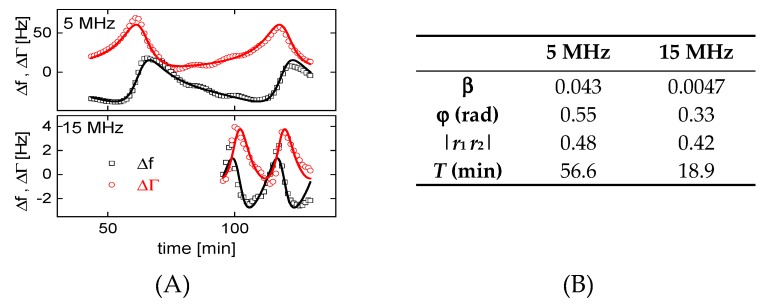
(**A**): Fits of Equation (11) to a subset of the data from Figure 7 (5 MHz and 15 MHz). The cuvette resonances are reproduced. (**B**) Corresponding fit parameters.

Figure 9A shows a fit of the model from Appendix A to these data. The fit function is

(11) $$\begin{array}{ll} \frac{\Delta f(t)+i\;\Delta \Gamma (t)}{f_{0}} & =\frac{i}{\pi \;Z_{q}}\left[Z_{shear}+\beta \;\exp(i\varphi )\;Z_{CW}(t)\right]\\ & =\frac{i}{\pi \;Z_{q}}[\sqrt{i\omega \rho \eta }+\beta \;\exp(i\varphi )\frac{Z_{CW,bulk}}{1-\left|r_{1}r_{2}\right|\exp\left(i\;\alpha \right)\;\exp\left(2\pi \;i\frac{t-t_{off}}{T}\right)}]. \end{array}$$

Within this model, the shear-wave impedance *Z_shear_* = (i ω ρ η)^1/2^ [42] and the compressional wave-impedance are additive in their effect on frequency and bandwidth. The parameter β accounts for the small amplitude of normal motion. While β depends on *U_N_*/*U_T_*, in principle, it was left as a fit parameter, here. The parameter φ accounts for a relative phase between bending and shear. As experiments shows, this phase is nonzero. *Z_CW,bulk_* = (*P* ρ)^1/2^ (with ρ the density and *P* the P-wave modulus) is the compressional-wave impedance of the semi-infinite medium. The denominator in the second line accounts for the multiple reflections. *R*_1_ and *r*_2_ are the complex reflectivities at the two surfaces of the cavity. (Only |*r*_1_ *r*_2_| is a fit parameter, because the phase α is not linearly independent from the phase −2π i *t_off_*/*T*). *T* is the time between to maxima of the cuvette resonances, *t_off_* is some offset in time. The fit parameters are shown in Figure 9B.

While both LDV and the measurements in liquids confirm the special role of flexural motion, it is worthwhile to remember that there is a second effect, which might cause problems with the fundamental mode. These are electric fringe fields combined with piezoelectric stiffening as described in chapter 14 in Reference [13]. Because energy trapping is least efficient on the fundamental mode, there may be wings in the distribution of the transverse amplitude, which extend to beyond the edge of the front electrode. In these portions of the plate, the piezoelectrically induced surface polarization is not compensated by charge inside the electrode. Electric fields may enter the liquid phase, which leads to an influence of the sample’s *electrical* impedance onto Δ*f* and ΔΓ. Reference [43] proves that possibility.

**Note added in proof:** One might think that compressional waves can be avoided by abandoning energy trapping. To this end, we tested a crystal with a front electrode covering the entire area and with the back electrode removed. One may drive a resonator without electrodes by exciting the vibration across an air gap. In the absence of energy trapping, the O-rings holding the resonator at the rim damp the resonance. That certainly is a disadvantage, but it can be dealt with. Subjecting this resonator to the experiment described in Section 5 (decreasing water levels), we found cuvette resonances similar to what is shown in Figure 7. The problem was not solved. Evidently, even this resonator did not vibrate in a pure thickness-shear mode. The reasons for this are unclear.

## 6. Conclusions and Outlook

Using LDV and a QCM in a liquid cell with a variable height of the liquid column, we find the effects of compressional waves to be stronger on the fundamental mode than on the overtones. LDV reports the ratio of normal to transverse motion. The coupled resonances are a measure of the strength of compressional-wave effects as experienced in QCM-based sensing. The normalized amplitude of flexural contributions decreases by a factor of two when going from the fundamental mode to the third overtone. The magnitude of the coupled resonance, on the other hand, decreases by a factor of 12. This work explains why QCM data from the fundamental mode often deviate from the expectations. It is a step towards full-fledged 3D modelling of QCM experiments.

## Figures and Tables

**Table 1:** Amplitudes of motion in the normal (*U_N_*) and the transverse direction (*U_T_*).

	14 mm Diameter (Dry)			14 mm Diameter (Wet)			25.4 mm diameter (Dry)		
*f* (MHz)	*U_N_* (nm)	*U_T_* (nm)	*U_N_*/*U_T_*(%)	*U_N_* (nm)	*U_T_* (nm)	*U_N_*/*U_T_*(%)	*U_N_* (nm)	*U_T_* (nm)	*U_N_*/*U_T_* (%)
**5**	1.38	124	1.1	0.29	18	1.6	1.10	84	1.3
**15**	0.20	35	0.6	0.027	5	0.5	0.22	22	1.0
**25**	0.12	15	0.8	0.016	3	0.5	0.20	15	1.3

**Table 2:** Distance (*D*) between the location of the maximum and the minimum of normal motion.

	14 mm Diameter (Dry)	14 mm Diameter (Wet)	25.4 mm Diameter (Dry)
*f* (MHz)	*D* (mm)	*D* (mm)	*D* (mm)
**5**	5.3	5.8	5.7
**15**	4.5	4.4	4.0
**25**	4.1	4.1	3.4

**Table 3:** Diameters of circles seen in the polar diagram (Figure 7C).

*f* (MHz)	Diameter of Circle (Hz)
5	60
15	5
25	5
35	1
45	3

**Figure A2:**
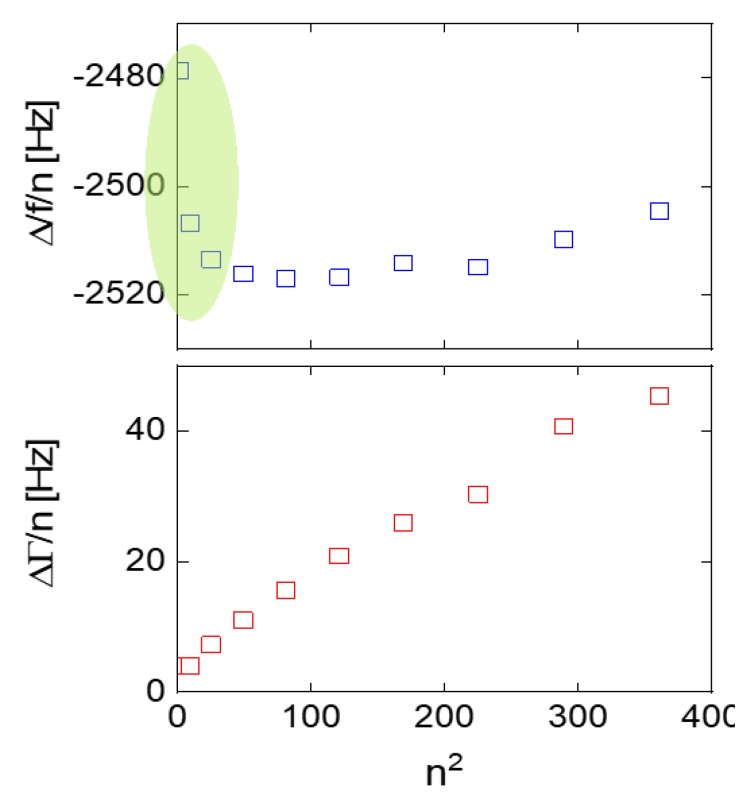
Overtone-normalized shifts of frequency and bandwidth obtained after depositing a polymer film with a thickness of about 500 nm onto the resonator plate. The area in green denotes values of Δ*f*/*n*, which are smaller than expected from the Sauerbrey relation. The difference goes back to flexural admixtures. Data from [47].

## Appendix A. Treatment of Compressional-Wave Effects in the Frame of the Small-Load Approximation

There is a general formulation of the small-load approximation, which covers both shear stress and normal stress at the resonator surface. It is based on the tensor form of the small-load approximation. The load impedance, *Z_L_*, is a ratio of stress to velocity. In its general form, the load impedance is a 3rd-rank tensor defined by

(A1) $$\sigma_{\alpha \beta }=Z_{L,\alpha \beta \gamma }\;v_{\gamma }.$$

σ is the stress tensor, v is the velocity. The tensor component related to shear has indices *xzx* (*z* the surface normal), the tensor component related to compression has indices *zzz*. All other tensor elements are set to zero. Following Section 6.1 in Reference [13], the complex frequency shift is

(A2) $$\Delta f+i\;\Delta \Gamma =\frac{i}{4\pi M_{eff}}\left(\int_{Surface}\frac{v_{x}^{2}\left(r_{S}\right)}{v_{ref}^{2}}Z_{L,xzx}\left(r_{S}\right)\;d^{2}r_{S}+\int_{Surface}\frac{v_{z}^{2}\left(r_{S}\right)}{v_{ref}^{2}}Z_{L,zzz}\left(r_{S}\right)\;d^{2}r_{S}\right).$$

*M_eff_* is an effective mass. *M_eff_* is equal to half the plate’s mass for the thickness-shear motion of a parallel plate, where the factor of 1/2 originates from the nodal planes inside the resonator. **r***_S_* is the location on the surface. v denotes a complex amplitude of a velocity (either tangentially, index *x*, or normal to the surface, index *z*). All velocities have been normalized to a reference velocity, v*_ref_*. The reference velocity is defined, such that the integral of v^2^/v*_ref_*^2^ equals unity (meaning that v^2^/v*_ref_*^2^ is a statistical weight).

If the small-load approximation holds, the transverse load and the normal load are additive in their consequences on the complex frequency shift. Using the language of the parallel-plate model, one may write

(A3) $$\frac{\Delta f+i\;\Delta \Gamma }{f_{0}}=\frac{i}{\pi \;Z_{q}}\left(Z_{L,xzx}+\beta \;Z_{L,zzz}\right).$$

*Z_L,xzx_* is the shear-wave impedance. *Z_L,zzz_* is the compressional-wave impedance. β is some numerical factor. Its value is an outcome of the integrations from Equation (A2). For the geometry under consideration, the transverse impedance, *Z_L,xzx_*, is given [42] as

(A4) $$Z_{L,xzx}=\sqrt{i\omega \rho \eta }.$$

With ω = 2π × 5 MHz, ρ = 1000 kg/m^3^, and η = 10^−3^ Pa s, one finds the absolute value of *Z_L,xzx_* to be 5.6 × 10^3^ kg/(m^2^s). If the liquid were truly semi-infinite even with regard to compressional waves (it is not), one would insert the bulk-compressional-wave impedance for *Z_L,zzz_* and write

(A5) $$Z_{L,zzz}\approx Z_{CW,bulk}=\sqrt{P\;\rho }=c_{CW}\;\rho .$$

The index *CW* denotes compressional waves. *P* is the P-wave modulus (close to the modulus of compression, much larger than the shear modulus). For quantitative evaluation, it is easier to use the second form in Equation (A5), where *c_CW_* is the speed of sound. With *c_CW_* ≈ 1000 m/s and ρ = 1000 kg/m^3^, one finds *Z_CW,bulk_* ≈ 10^6^ kg/(m^2^s).

*Z_L,zzz_* is computed similarly to how optical fields are computed for planar optical cavities (also: “Fabry-Perot filters”, see [44]). The total impedance at the resonator surface is a geometric series of the form

(A6) $$Z_{L,zzz}=Z_{CW,bulk}\sum_{n=0}^{\infty }{\left[r_{1}\;r_{2}\;\exp\left(2\;i\;k_{C}\;d_{liq}\right)\right]}^{n}=Z_{CW,bulk}\frac{1}{1-r_{1}\;r_{2}\exp\left(2\;i\;k_{C}\;d_{liq}\right)}.$$

The complex parameters *r*_1_ *and r*_2_ are the reflectivities at the two surfaces of the cavity, *k_C_* is the wavenumber, *d_liq_* is the thickness of the liquid cell, and 2 *k_C_ d_liq_* is the phase accumulated per round trip (not taking reflections into account).

We assume an evaporation rate that is constant in time (about 1 mm/day), and define the time *T* as the time elapsed while the air/water interface approaches the resonator surface by half a wavelength (the time between two peaks in Figure 7). We find *T* ≈ 1 h for the 5 MHz data and *T* ≈ 20 min for the 15 MHz data. A constant evaporation rate leads to

(A7) $$\begin{array}{ll} Z_{bend}\left(t\right) & \approx Z_{L,zzz}\left(t\right)\approx \frac{Z_{CW,bulk}}{1-r_{1}r_{2}\;\exp\left(2\pi \;i\frac{t-t_{off}}{T}\right)}\\ & =\frac{Z_{CW,bulk}}{1-\left|r_{1}r_{2}\right|\exp\left(i\;\alpha \right)\;\exp\left(2\pi \;i\frac{t}{T}\right)\;\exp\left(-2\pi \;i\frac{t_{off}}{T}\right)}\begin{array}{c} . \end{array} \end{array}$$

*t_off_* is some offset in time. In the second line, *r*_1_ *r*_2_ was as expressed as |*r*_1_ *r*_2_|exp(i α) with the phase α. The phase cannot be determined from the fit, because it is not linearly independent from the offset in time, *t_off_*. Equation (A7) inserted into Equation (A2) leads to the fit function (Equation (11)) used to produce the solid lines in Figure 9A.

## Appendix B. Estimation of the Transverse Amplitude from the Electrical Power

All resonators were driven with a power of +15 dBm. Converted to SI units, this corresponds to a time-averaged power of *P_avg_* = 10^15/10^ mW = 31.6 mW. The amplitude *I*_0_ of the current follows from the relations

(A8) $$P_{avg}=\frac{1}{2}\;R_{1}\;I_{0}^{2},\;I_{0}=\sqrt{2\frac{P_{avg}}{R_{1}}}.$$

The index 0 denotes an amplitude. *R*_1_ is the motional resistance as determined from impedance analysis. The amplitude of the velocity at the resonator surface, v*_T_*, is given as [16]

(A9) $$v_{T}=\frac{d_{q}}{2\;A_{eff}\;e_{26}}I_{0}.$$

*d_q_* = 330 µm is the thickness of the plate. *e*_26_ = 9.65 × 10^−2^ C/m^2^ is the piezoelectric stress coefficient. The parameter *A_eff_* is the effective area, calculated from Equation 7.4.7 in [13] as

(A10) $$A_{eff}=\frac{n\;\pi }{32\;Z_{q}\;d_{26}^{2}\;f_{0}^{2}}\frac{1}{Q\;R_{1}}.$$

*d*_26_ = 3.1 × 10^−12^ m/V is the piezoelectric strain coefficient. *Q* is the quality factor. From the amplitude of the velocity, v*_T_*, the amplitude of the displacement, *U_T_*, follows as

(A11) $$U_{T}=\frac{v_{T}}{\omega }.$$

The outcome of these calculations is summarized in Table A1.

A side remark: At a drive level of 15 dBm, one notices a slight increase of the resonance frequency in response to the large amplitude. The effect is caused by a small elastic anharmonicity (and is well known [45]). While we did not study matter in detail, we do not expect a change of the vibration pattern caused by large amplitudes.

**Table A1:** Parameters (determined by impedance analysis undertaken with the network analyzer E5100 from Agilent) entering the estimation of the transverse amplitudes and resulting transverse amplitudes in Table 1.

	14 mm Diameter (Dry)			14 mm Diameter (Wet)			25.4 mm Diameter (Dry)		
*f* (MHz)	*A_eff_* (mm²)	*R*_1_ (Ω)	*U_T_* (nm)	*A_eff_* (mm²)	*R*_1_ (Ω)	*U_T_* (nm)	*A_eff_* (mm²)	*R*_1_ (Ω)	*U_T_* (nm)
**5**	25	19	124	46	280	18	41	16	84
**15**	27	24	35	38	590	5	32	42	22
**25**	25	52	15	30	980	3	25	52	15

## Appendix C. Zernike Decomposition of the Operating Deflection Shapes

In order to extract quantitative parameters from each ODS for comparison between the different experimental configurations, a Zernike decomposition was performed. Figure A1 documents this process for the mode shown in Figure 3C. Zernike modes with indices higher than 78 and modes with amplitudes lower than 10% of the largest amplitude were discarded (red lines).

## Appendix D. Assessment of the Magnitude of Flexural Admixtures from Experiments in Air

Flexural admixtures not only affect measurements in liquids (because of compressional waves), but also measurements of the mass of a film in the dry following Sauerbrey [46]. We briefly go through the argument. The matter is in more depth described in Section 6.1.4 in Reference [13].

Start from the classical formula for the resonance frequency of a harmonic oscillator, which is

(A12) $$\omega =\sqrt{\frac{\kappa }{M}}.$$

This formula holds for discrete objects. κ is a spring constant, *M* is a mass. Applied to elastic bodies vibrating in some vibration mode, equivalent parameters must be (and can be) defined. The definition of κ is unessential here. Write the mass as *M_q_* + *m_f_* with *M_q_* a “modal mass” of the resonator and *m_f_* the mass of a film deposited on the resonator surface.

Assuming *m_f_* << *M_q_*, one may write

(A13) $$\begin{array}{ll} \Delta f=\frac{\Delta \omega }{2\pi } & =\frac{1}{2\pi }\left(\sqrt{\frac{\kappa }{M_{q}+m_{f}}}-\sqrt{\frac{\kappa }{M_{q}}}\right)=\frac{1}{2\pi }\sqrt{\frac{\kappa }{M_{q}}}\left(\frac{1}{\sqrt{1+m_{f}/M_{q}}}-1\right)\\ & \approx \frac{1}{2\pi }\sqrt{\frac{\kappa }{M_{q}}}\left[1-\frac{m_{f}}{2\;M_{q}}-1\right]=-f\left(\frac{m_{f}}{2\;M_{q}}\right). \end{array}$$

Taylor expansion was applied ((1 + ε)^−1/2^ ≈ 1 − ε/2). At first glance, Equation (A13) appears to be in conflict with the Sauerbrey equation [46], which states that

(A14) $$\frac{\Delta f}{f}=-\frac{m_{f}}{m_{q}}.$$

*m_q_* here is the mass of the resonator plate. The solution to this puzzle is that the modal mass, *M_q_*, is only half the mass of the resonator plate, *m_q_*. This is because the parallel plate contains nodal planes. Averaging the square of the velocity distribution over the volume of the plate (the key step in the derivation of the modal mass) produces a factor of 1/2 (meaning that *M_q_* = *m_q_*/2).

This argument, however, assumes a thickness-shear deformation. Flexural admixtures violate this assumption because the flexural motion does not depend on *z*. With flexural admixtures present, the modal mass increases and the overtone-normalized frequency shift Δ*f*/*n* therefore decreases.

That is demonstrated in Figure A2 (data from [47]). Δ*f*/*n* and ΔΓ/*n* are plotted versus *n*^2^ (rather than *n*), because the slope in such plots is related to the film’s viscoelastic compliance. Importantly, flexural admixtures affect the frequency shift not only at the fundamental mode, but also at overtones 3 and 5. The main text suggested that flexural admixtures were mostly a problem at the fundamental mode. In this example, flexural admixtures are a problem on overtones 1 to 5 (and even 7).

## Appendix E. The LDV Instrument Used to Measure Transverse Components of the Displacement

In order to measure the transverse displacement, the resonator needs to be tilted and the LDV detection has to rely on scattering rather than reflection. The LDV setup is sketched in Figure A3. For the given arrangement, the transverse or tangential component of the displacement is estimated by dividing the measured absolute values by cos (55°).
